# Improving low fruit and vegetable intake in children: Findings from a system dynamics, community group model building study

**DOI:** 10.1371/journal.pone.0221107

**Published:** 2019-08-15

**Authors:** Sarah Gerritsen, Ana Renker-Darby, Sophia Harré, David Rees, Debbie A. Raroa, Michele Eickstaedt, Zaynel Sushil, Kerry Allan, Ann E. Bartos, Wilma E. Waterlander, Boyd Swinburn

**Affiliations:** 1 School of Population Health, University of Auckland, Auckland, New Zealand; 2 Synergia Ltd., Auckland, New Zealand; 3 Healthy Families Waitakere, Auckland, New Zealand; 4 School of Environment, University of Auckland, Auckland, New Zealand; 5 Department of Public Health, Amsterdam UMC, University of Amsterdam, Amsterdam, The Netherlands; University College London, UNITED KINGDOM

## Abstract

Many children globally do not meet government guidelines for daily fruit and vegetable intake, and in New Zealand, adherence to the vegetable intake recommendation is declining. This study aimed to identify systemic barriers to children meeting fruit and vegetable (FV) guidelines and generate sustainable actions within a local community to improve children’s FV intake. A qualitative system dynamics method of community group model building was used. The research team partnered with Healthy Families Waitākere, a Ministry of Health funded prevention initiative, to recruit 17 participants (including students, parents, teachers, community leaders, local retailers and health promoters) from a low-income, ethnically-diverse community in West Auckland, New Zealand. Three group model building workshops were held during which a systems map was created and used to identify actions by considering causal pathways and reinforcing loops in the system. Barriers to children’s FV intake identified by participants were the saturation of fast-food outlets in the community and ubiquitous marketing of these products, the high cost of fresh produce compared to fast food, and parents having little time for food preparation plus declining cooking skills and knowledge. Several actions to improve children’s FV intake by improving the local food environment were identified, which will be co-designed further and tested by a collaborative group involving community leaders. This project highlights the effectiveness of group model building for engaging a local community in systems change to improve child nutrition, and supplies a blueprint for future qualitative system dynamics research.

## Introduction

Adequate fruit and vegetable (FV) consumption are crucial for children’s health. The current New Zealand (NZ) dietary guidelines recommend that young children under five years should eat at least two servings of vegetables and two servings of fruit per day, while children older than five years should eat at least three servings of vegetables and two of fruit [1]. The latest NZ Health Survey found 51.4% of 2–14 year-olds met the recommended serves of vegetables per day, and 72.4% consumed adequate fruit [2]. Over the past five years, the proportion of children meeting the vegetable recommendation has declined (from 58% to 51%), and fruit intake remains unchanged [2]. FV intake is even lower in the Auckland region, which is the most populous region of NZ, with 42% of Auckland children meeting the FV recommendations [3]. Vegetable intake is lowest among non-European children and in neighbourhoods of high deprivation [2]. Only two out of every five children living in the 20% most deprived neighbourhoods meet the vegetable intake recommendation [2].

These trends are of public health concern. FV consumption in childhood provides valuable nutrients for growth and development, strengthens immunity, and aids digestion [1,4]. Low FV intake is associated with children’s low academic achievement [5]. In addition, children who consume sufficient FV have a lower risk of obesity and obesity-related illness [6], and they are more likely to establish a healthy dietary pattern for life [7, 8]. Malnutrition in all its forms (which includes macro- and micro-nutrient deficiencies, overweight and obesity) is now the largest contributor to disability-adjusted life years (DALYs) lost globally and in New Zealand [9].

Healthy Families New Zealand is a Ministry of Health funded initiative, operating in ten locations across Aotearoa with higher than average rates of preventable chronic disease and/or deprivation [10]. Healthy Families Waitākere works to strengthen the prevention system in West Auckland, through supporting community leaders to think differently about the underlying causes of poor health, and identify systems change which enables healthier choices in places where we spend our time, including; schools, workplaces, places of worship, marae, community spaces and more. By taking a systems approach to reducing risk factors of preventable chronic disease, the approach aims to improve health outcomes and increase health equity through key focus areas; improved nutrition, physical activity and mental health, smoke-free and reduced alcohol-related harm. Healthy Families Waitākere is delivered by Sport Waitākere, working together to create sustainable, healthy change in our communities.

System dynamics (SD) provides a structured approach for tackling ‘wicked’ problems, such as malnutrition in all its forms [11, 12]. SD requires multi-disciplinary teams and can include community participatory approaches to assist with problem structuring, system conceptualization, and capacity building [13]. In the qualitative use of SD, causal loop diagrams (CLD) are developed with community participants to understand how key elements in a system interact and feedback upon each other to produce the behaviour of concern [14]. This SD approach has been found to be highly effective for engaging communities in developing ‘bottom-up’ solutions and exploring barriers to action [13]. Actions that are acceptable to the community and sustainable within the existing system can then be developed. CLDs assist community stakeholders to express their understanding of the system surrounding an issue, regardless of prior experience with systems thinking [13]. SD methods can engage community members to become active leaders and promote ownership of the actions created through the process [13]. The application of system dynamics to public health nutrition is relatively new. SD has been used in public health research to examine obesity in communities [14–17], interviewing experts on the determinants of healthy eating [18], and developing food-related policy making [19,20]. Previous research in this area has called for case-studies to provide insight into particular parts of the food system in order to support the development of actions [21].

This paper presents findings from workshops held with a low-income ethnically-diverse community in the Healthy Families Waitākere (HFW), West Auckland, site. Participants were led through a structured SD method to create a CLD.

The research aimed to identify:

- current systemic barriers to meeting the fruit and vegetable (FV) guideline among 2–14 year old children; and
- community-developed options for systemic actions which would improve children’s nutrition by increasing FV intake.

## Materials and methods

The Consolidated Criteria for Reporting Qualitative Research (COREQ) checklist [22] has been followed (S1 Table).

### Research team

The academic researchers partnered with Healthy Families Waitākere (HFW) to develop the study design, recruitment procedures and to assist with interpretation of findings. HFW has extensive knowledge of, and networks within, West Auckland communities and an expressed commitment to catalysing action on this issue beyond the end of the project. The study team comprised public health nutrition academics (BS, SG, WW) and students (SH, ED and AR), a system dynamics consultant (DR), a food systems academic (AB), and HFW staff with a variety of areas of trained expertise (KA, DAR, ME, ZS).

### Study design and ethical approval

This study used a SD method called group model building (GMB), which is a participatory method of building a systems model involving a large group of typically 15–25 people [13]. GMB was used to engage community members in thinking about causal pathways and reinforcing loops within their food system and to design sustainable and acceptable actions to increase children’s FV consumption. This study was conducted according to the guidelines laid down in the Declaration of Helsinki. All participants were aged 16 years or over and provided written informed consent to take part in this study. Ethical approval was granted by the University of Auckland’s Human Participants Ethics Committee on 1 March 2018 (ref: 020684).

### Setting and participant selection

A neighbourhood community within the HFW location was selected as the study setting because its population was relatively young, with low median household income (decile 3 schools) and a high proportion of people identifying with Pacific or Asian ethnicities; all of which are independent risk factors for low fruit and vegetable consumption [2]. The predominant household in the community is young families. There are 11 schools and early education services in the area (only 1 of which is involved in the NZ Government’s Fruit in Schools programme). There is one supermarket, several take-away and fast-food outlets, and several smaller corner grocery stores, some of which sell FV.

Due to HFW’s knowledge and strong relationships within the location, participant selection was led by HFW in collaboration with the project’s research assistant (based onsite at the HFW office). Participants were selected to represent the main sectors of the local food system including local retailers, health promoters, schools and the wider community, with a minimum of two from each of these sectors. Secondary school students were included if they were over 16 years of age. The majority of participants were recruited through HFW existing networks. Once invited by HFW, a number of participants also recruited from within their own networks (snowballing). Community flyers, notices in school newsletters, and in-person visits to food retailers were largely unsuccessful in recruiting participants.

### Data collection and tasks at the workshops

Data were collected during 3-hour GMB workshops held over three consecutive weeks in the evening at a local school in the community (9 hours total). These workshops were led by a session facilitator (SG) and modeller (DR) from the study team, following GMB structure and principles [13, 23–25]. A HFW staff member (DAR) acted as the ‘relationship facilitator,’ advocating on behalf of the community members with the research team and on behalf of the research team with the community members [24].

Table 1 details the tasks undertaken during each workshop. Each workshop began with a welcome and blessing from local kaumātua (Māori elder) and ‘whakawhanaungatanga’ (Māori tikanga/process for establishing relationships and getting to know each other) which included sharing a meal together. Participants were then placed into small groups of four to five at a table by the ‘relationship facilitator’, with each table including a facilitator from the research team. These small groups were rearranged each week with participants at each table spanning the different sectors (health promotion, retail, school, community), where possible. The session facilitator and modeller led various tasks described in Table 1 while table facilitators guided small group discussions and ensured the quality of the outputs (14). The workshops were not recorded, but one of the researchers wrote down quotes from participants during whole group discussions. At the conclusion of the third workshop, each participant was given a $100 voucher to thank them for their participation.

**Table 1:** Workshop tasks.

Workshop	Task name	Task description
Workshop One	Children’s FV consumption	Explanation of issue: declining FV intake in children and how it relates to this community
	Graphs over time (GOT) [23, p23)	Working individually, participants graphed variables in their community that were changing over time that they perceived contributed to children’s declining FV consumption
	Introduction to systems thinking	Modeller explained the concept of systems thinking (including the components of systems such as causal connections between variables, mental models and values)
	Causes and consequences	Participants brainstormed the causes and consequences of children eating FV as well as children not eating FV Participants were encouraged to make links between causes and consequences to form beginnings of causal loops
Workshop Two	Presenting themes of GOT and linear map	Session facilitator presented the themes from the graphs over time and the composite linear map showing causes and consequences Participants were invited to comment, criticise, or make changes to both
	Introduction to CLDs	Modeller introduced the principles of CLDs Modeller presented the initial CLD built from the causes and consequences map
	Building CLDs in table groups [23, p30]	Participants created CLDs in their table groups, building on the CLD that the modeller presented
Workshop Three	Presenting summary CLD	Session facilitator presented the summary CLD In their table groups, participants discussed any changes they wanted to make to the diagram
	Identifying points on CLD for interventions	Participants identified points on the CLD for potential influence and action Top five actions were chosen at each table group and shared with the whole workshop group
	Spheres of influence	Participants identified areas of their life over which they had influence, ranging from individual life to the community to greater New Zealand (S3 Fig)
	Priority matrix	At their table groups, participants plotted their top five actions on a priority matrix (S4 Fig)

Acronyms: FV, fruit and vegetable; CLD, causal loop diagram, GOT, graphs over time

### Data analysis and refinement of the systems map between workshops

Following the first workshop, the research team summarised the variables from the graphs over time task into themes (S1 Fig) and used these along with the variables from the causes and consequences exercise to form a single linear map showing causal direction between variables (S2 Fig). This map did not contain any feedback loops at this stage. The modeller then used the linear map to build an initial causal loop diagram (CLD) to illustrate connections between variables.

After the second workshop, the research team refined the CLDs produced by each table group and integrated them into one diagram using Vensim (SD modelling software, Vensim.com). Some variables were discarded if irrelevant to the research purpose, while others were added if they were discussed during the workshop but not written down. This refinement process ensured that the final systems map captured the stories emerging from the workshop, and was checked with participants at the start of the following workshop.

After the third and final workshop, the research team analysed the two main outputs of the research; the systems map and the list of proposed community actions. Changes to the systems map which were suggested during the final workshop were integrated. The systems map was then tidied using Vensim and checked to ensure that the main stories from the discussions at each workshop were captured. This process included adding variables or links that were discussed but not captured in the initial CLDs. Then the systems map was simplified using the principles of SD [13], by amending the naming of variables as well as adding variables and editing the polarity of arrows to ensure that the final systems map conveyed participants’ intended meanings. The list of proposed actions were also tidied and organised into themes.

The final CLD was presented and reconfirmed with workshop participants approximately one month later, and their willingness to be involved in further co-design of the actions with HFW was collected.

## Results

A total of 17 community members participated in the three workshops. One participant only attended the first workshop, and two other participants attended two of the three workshops. The remaining 14 participants attended all workshops. Table 2 presents a description of workshop participants, showing diversity in age, gender, and roles. All main ethnic groups in the community (Māori, Pacific, Asian and NZ European) were represented, with over half of participants identifying as Māori or Pacific, but the ethnicity of individuals has been excluded from Table 2 to assist with confidentiality.

**Table 2:** Description of the workshop participants.

	Sector	Organisation/role	Gender	Age range
**1**	Health promotion	Public health nurse educator	Female	25–40
**2**	Health promotion	Community physical activity coordinator	Male	25–40
**3**	Retail	Supermarket employee	Female	25–40
**4**	Retail	Caterer and school tuckshop manager	Female	40+
**5**	School	High school student	Male	16–25
**6**	School	High school student	Male	16–25
**7**	School	High school sport co-ordinator	Male	40+
**8**	School / Community	School board of trustee member and local elder (kaumatua)	Female	40+
**9**	Community	Former high school student	Female	16–25
**10**	Community	Community board trustee	Female	25–40
**11**	Community	Community garden facilitator	Female	40+
**12**	Community	Community centre administrator	Female	40+
**13**	Community	Community centre facilitator	Female	40+
**14**	Community	Parent	Female	40+
**15**	Community	Parent	Female	25–40
**16**	Community	Parent	Female	25–40
**17**	Community	Parent	Female	25–40

**Fig 1:**
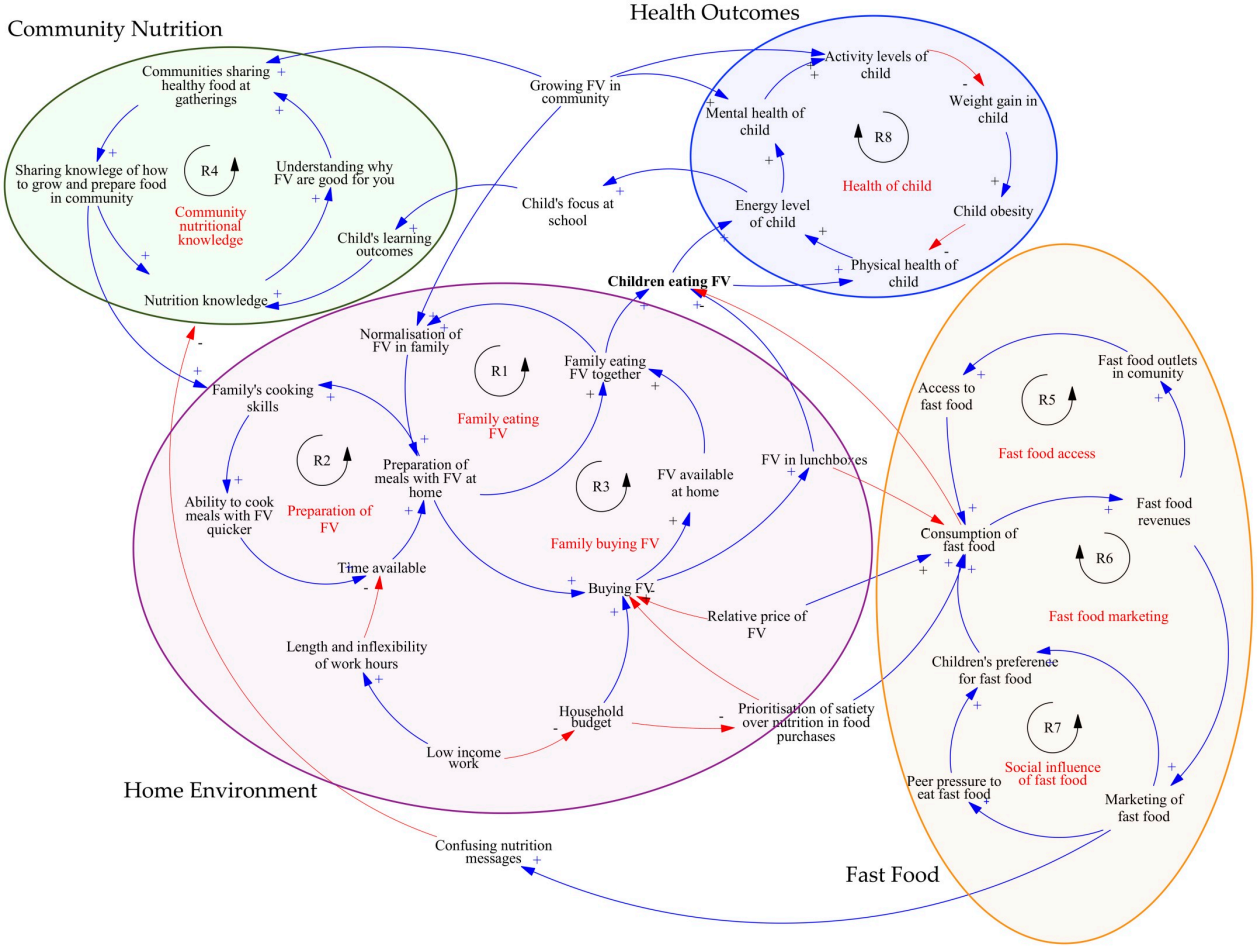
Community food system map (causal loop diagram). A causal loop diagram (CLD) uses arrows to indicate a causal relationship between variables in a system [25]. The CLDs created in this study describe the causal connection between variables that workshop participants deemed to be important, highlighting the feedback processes that determine system behaviour [25]. A blue arrow with a plus symbol shows that the two variables move in the same direction; if the variable at the tail of the arrow increases or decreases, the variable at the head also increases or decreases, respectively. A red arrow with a minus symbol indicates that the two variables move in opposite directions: if the variable at the arrow tail increases, the variable at the head decreases (and vice versa). The causal loops within the CLD illustrate the way in which a chain effect of a cause can be traced through a set of related variables, back to the original cause [25]. All causal loops in this systems map are reinforcing, indicated with an R in the diagram, which means that they perpetuate either a vicious or virtuous cycle [25].

Fig 1 shows the final systems map of the local community food system which was iteratively constructed across the three workshops. Four subsystems in the food system are circled and shaded: The home environment, Fast food, Community nutrition and Health outcomes. Workshop participants’ thinking behind the variables is explained below, ordered by subsystem. Reinforcing loops are indicated with an R (Fig 1).

### Subsystem 1: The home environment

The first subsystem centred on the home environment (see R1, R2 and R3 in Fig 1). Participants noted children ate more FV if their families were eating more FV. A major feedback structure identified was the normalisation of FV at home (Fig 1, R1), which increased when the family ate FV together, with adults role-modelling healthy eating. Another important reinforcing loop in this subsystem was the preparation of FV and development of cooking skills (Fig 1, R2). Participants noted that cooking skills and the ability to prepare meals with FV increased when families did this more often.

Participants observed that the ability to quickly prepare meals with FV improved with practice. However this cycle was difficult to start due to limited time available. Parents having limited available time for food preparation was thought to be caused by low income work, with multiple-jobs and inflexible hours reported by workshop participants as common in their community. Low-income work also meant many families had low household budgets and were, therefore, more likely to prioritise satiety over including FV for nutrition in their food purchases.

### Subsystem 2: Fast food

The prioritisation of satiety over nutrition when making food purchases also meant that families were more likely to buy fast food (see R5, R6 in Fig 1). Participants stated fast food was replacing FV in children’s diet. Fast food was defined by participants as food that was high in sugar, salt, and saturated fat. Participants noted fast food was very accessible in their community, particularly McDonald’s, pizza, and hot chips. The first feedback loop (Fig 1, R5) focused on the increasing saturation of fast food outlets in the community, including outside schools. Participants noted this trend was driven by the growing number of fast food outlets, caused by the the increased consumption of fast food, which resulted in a ‘vicious’ perpetuating cycle. Increased marketing and availability of fast food (Fig 1, R6 and R7) was identified as a recent change in their community and an important determinant of children consuming fast food. Participants said that this marketing contributed to peer pressure to eat fast food.

*“How do you change what is sold right outside the school? I have a lot of respect for the shop owners*—*we all grew up knowing them*—*but what they’re selling is not good for our kids*.*”*

*“When I open the Uber Eats app in this neighbourhood there are 117 places that can deliver me food in 20 minutes and none of them are a healthy choice*.*”*

*“My teenage son gets Uber Eats delivered to his bedroom window*—*how can I compete with that?”*

### Subsystem 3: Community nutrition

Workshop participants noted fast-food marketing and the inaccurate or misleading labels on packaged foods resulted in confusing nutrition messages. These variables led into the third subsystem on the CLD, comprised of one reinforcing loop centred on community nutritional knowledge (Fig 1, R4). Participants identified the effect of community practices and norms on children’s FV intake, mentioning food prepared for community gatherings at church and on the marae (Māori meeting grounds) which could be either positive or negative in aiding FV intake. Variables included the growing and sharing of healthy food in the community, as well as the importance of understanding why FV are healthy.

*“We need to make eating veg a norm*.*”*

### Subsystem 4: Health outcomes

Participants identified when children were involved with growing FV in the community, their mental health improved and they were more physically active (Fig 1, R8). All participants mentioned the likely outcome of decreased diet quality when children did not eat FV, and knew the consequences of a poor diet could be diabetes, cardiovascular disease and premature death. Participants also mentioned that eating FV had positive results beyond the physical aspects of a child’s health, including increased focus at school and the ability to learn more about the positive effects of FV (Fig 1, R8).

### Actions proposed by the participants

Participants were asked to offer potential actions based on the concept of leverage in system dynamics (that is, identifying actions which change or break a causal loop in order to alter a variable of interest [25]). The participants then prioritized the actions according to perceived feasibility and impact (S4 Fig). Eighteen actions were described by the group, listed in Table 3 according to the CLD subsystem (Fig 1) that the participants identified the action would influence.

**Table 3:** Actions proposed by workshop participants and their level of change. [26].

Subsystem	Action	Level of change
**Home environment**	Children’s books about FV	Individual behaviour change—nutrition education
	FV delivery truck	Community food environment
	FV posters in community	Individual behaviour change—public awareness campaigns
	Cooking classes at community centre	Individual behaviour change—skill building
	Gardening workshop at community centre	Individual behaviour change—skill building
	Holistic wellbeing workshop at community centre	Individual behaviour change—nutrition education
**Fast food**	Community ‘license’ or visable sticker for healthy food outlets	Community food environment
	Raising awareness of fast food industry	Individual behaviour change—public awareness campaigns
	Board of trustees healthy school food policies	School food environment
	Reduce fast-food advertising	[Action not developed in detail by participants]
	School trips to supermarket to learn about FV	Individual behaviour change—nutrition education
**Community nutritional knowledge**	Sharing healthy food at church and on the marae (Māori meeting grounds)	Community food environment
	Expanding free FV to all schools	School food environment
	Seed vault at community centre	Individual behaviour change—skill building
	‘Healthy living bus’ at community events	Individual behaviour change—nutrition education

Participants noted the need to counter the fast food industry with tactics similar to fast food marketing in their community:

*“Two things: affordability and access*. *If everything is being ubered to your house*, *we need to be there with fruit and veg too*. *Like a milk truck but veggies*.*”*

*“Yes*, *a food truck*. *We all know we need fruit and veg everyday*, *so why not have it delivered like milk used to be?”*

*“Put a sticker in the window of shops that are healthy: [Community’s name] Pride! Let’s build on the community spirit here*, *and if you’re not healthy–if you don’t serve FV–then you don’t get a sticker*.*”*

Participants were energised to continue their planning for actions with HFW following the final workshop.

*“There’s definitely a lot of passion in this community*, *and then everyone will get on to it*.*”*

## Discussion

This paper presents the results of collaborative research conducted alongside a Ministry of Health funded prevention initiative (HFW) with members of an ethnically-diverse, low-income community in Auckland, NZ. Using SD methods, this research enabled participants to identify several systemic barriers to children meeting the FV guidelines and produce a list of actions they proposed would increase children’s FV intake. This study builds on the emerging use of SD in public health literature to apply SD in the area of child nutrition.

Most notable in this research was the lack of alignment between the barriers and enablers (or levers) identified in the CLD (Fig 1) and the community actions proposed (Table 3). The GMB process was successful at shifting participants’ thinking so they were able to see and understand the systemic barriers preventing children from eating sufficient FV, but it was much more difficult for participants to see the actions that could potentially influence the deeper systemic issues of culture, social norms, and the underlying goals of the system. Participants tended to revert to traditional, individual-focused health promotion interventions, rather than engaging with the causal loops in their systems map to brainstorm actions based on how they could intervene or change the intensity or direction of the causal loops. Developing an understanding of systems levers is potentially a longer journey than we allowed time for in the three workshops, and should be anticipated in future community research. In continuation of this project, HFW is working with a subgroup of workshop participants using the principles of co-design to prototype and test the actions proposed. This subsequent additional phase could be considered for future GMB projects to further develop, implement and test actions proposed by the community [27].

The main barriers and suggested solutions proposed by the community are now considered in detail alongside other studies on the determinants of children’s FV intake and the effectiveness of actions taken to improve FV intake.

### The density of fast-food outlets and fast-food marketing

Workshop participants observed a high density and rapid increase over the past few years in the number of fast-food outlets in their community, resulting in children consuming more fast-food which displaced FV in their diet. National studies on the distribution of fast-food outlets in NZ found that the relative density of unhealthy food outlets were significantly higher in the most deprived areas of NZ compared to the least deprived [28,29]. Participants also commented on the close proximity of fast-food outlets to schools in their community. NZ data shows there are significantly more convenience stores, fast-food and takeaway outlets around the most deprived urban schools in comparison to the least deprived schools [28]. A related issue mentioned by participants was the proliferation of online food ordering platforms (e.g. Uber Eats) which increase the availability and accessibility of fast-food. Currently there is no national or council-level regulation on the number of fast-food outlets permitted in a community or school zone in NZ, but this has been suggested elsewhere as a potential solution to the proliferation of fast-food vendors in low-income areas [29].

Workshop participants also noted children in their community were surrounded by fast-food marketing, which increased children’s preference and purchasing of those food products; an observation supported in the international literature [30,31]. In NZ, marketing to children is regulated by an industry group, the Advertising Standards Authority [32]. The Children and Young People’s Advertising Code states that advertisements for occasional foods (as defined by the Food and Beverage Classification system) should not target children or be placed in media where children form a significant part (over 25%) of the audience [33]. However, national data show that children are regularly targeted by fast-food marketing, on television, in magazines aimed at adolescents, through company websites, on social media, and through the sponsorship of children’s sports and sport clubs [33]. Furthermore, there is a greater density of unhealthy food advertisements around the most deprived schools in comparison to the least deprived schools, showing that children in low socioeconomic areas are inequitably targeted by fast-food marketing [30].

To address this issue, participants acknowledged that there needs to be a reduction in fast-food marketing, but they could not go into detail about how this could be achieved. This suggests that this issue may be beyond the community’s sphere of influence and require structural change at a national level. This sentiment was noted in the Healthy Families NZ Summative Evaluation, which noted that there were systemic barriers to reducing preventable chronic disease risk factors (e.g. poverty) that must be addressed by the government [10].

### School food policies and programmes

Participants suggested one way to limit children’s access to fast-food was through school food policies and programmes. International evidence suggests nutrition guidelines and price interventions focused on healthier foods are effective in improving the school food environment and students’ dietary intake [34]. In NZ, the Ministry of Education’s National Administration Guidelines state schools should “promote healthy food and nutrition for all students” [35]. Individual school’s Board of Trustees (parents and community members) set their own school policies. A survey of schools in 2016 found 38.5% of primary and 44.8% of secondary schools had a food policy [30]. Few of these policies addressed students leaving school grounds for lunch, standards for food and beverages brought from home, or steps to promote healthy food choices in the canteen or tuckshop [30].

Workshop participants suggested providing free fruit and vegetables in schools to improve children’s nutrition. International systematic reviews and meta-analyses show that FV provision programmes do not usually increase children’s overall FV consumption [36,37]. However, there is a lack of research around the long-term effects of these programmes in promoting a healthy diet and increasing taste preferences for FV. Previous research has found repeated taste exposure, particularly to vegetables in early childhood, is the most effective action to increase FV intake. In NZ, the Ministry of Health currently funds one piece of fruit or vegetable daily for Year 1 to 8 students in decile 1 and 2 schools (the areas of highest socioeconomic deprivation) [38]. Nearly all of the schools in the community involved in this study were decile 3, and therefore miss out on the programme. The 2018 evaluation of Fruit (and Vegetables) In Schools concluded that the programme was successful in promoting a healthy school environment and encouraging the school to use other health promotion resources [38].

Additionally, principals involved in the evaluation noted children who did not have breakfast before school and/or came to school with no lunch were able to be given some fruit, rather than go hungry. The evaluation recommended the programme be expanded to include decile 3 and 4 schools, and the participants in this study would welcome Fruit (and Vegetables) In Schools in their community. With more than 50% of NZ children not meeting the daily recommended intake of FV [2], the need to increase FV intake clearly extends beyond primary school children living in the poorest 20% of communities. Therefore an expansion of the Fruit (and Vegetables) In Schools programme to include early education services and a greater number of schools would appear warranted.

### Small food budgets and lack of time for food preparation in low-income families

Participants noted that low-income families often had to prioritise satiety over nutrition in food purchasing, which resulted in them buying less FV (Fig 1). FV was often perceived as high-cost in relation to fast-food because it was not thought to be as filling (energy-dense). Although the systemic barrier of low-income work was highlighted by community participants as the main driver of small food budgets and not enough money to purchase FV, no actions were proposed that would mitigate or lessen this barrier. Modelling of typical NZ diets found that eating a healthy diet is on average more expensive than eating an unhealthy diet, particularly when takeaway foods were included [39]. While it was possible to eat a healthy diet for the same price as an unhealthy diet, this required more time and cooking skills to achieve [39]. These barriers were also recognised by workshop participants, who confirmed that a lack of time and cooking skills were important factors in children’s low FV consumption. Several solutions were proposed by workshop participants designed to increase the cooking skills and nutritional knowledge of their community.

### Improving community nutrition

When brainstorming actions to improve the home and community food environment, participants focused predominantly on individual behaviour change. Suggested actions included FV posters in the community, wellbeing workshops, a ‘healthy living bus’ at community events distributing health information, and cooking and gardening classes at the community centre (Table 3). The research evidence on these actions is mixed. To date, evaluations of school and community cooking and gardening classes have been of varying quality, with different tools and limited follow-up to measure change so the likely effect on community nutrition is unclear [40]. However, in comparison to standard curriculum-based nutrition education (for example, a lesson in the classroom on the nutrients in fruits), experiential learning in a school garden has been found to have a greater influence on children’s consumption/energy intake and increasing nutritional knowledge [41]. Children are more willing to try foods after involvement in the growing, preparation and/or cooking of that food, and consumption and skills increases in the short term [42,43]. A comprehensive approach to improving food preparation, knowledge and cooking skills, such as that found in Jamie’s Ministry of Food programme [44], appears to be the most promising for improving community dietary quality.

Posters, brochures and other forms of social communication to share nutritional knowledge have been found to be effective if they can stimulate demand, publicise new options or assist with building social movements, but usually only when combined with wider environmental change [45, 46]. Health promotion in NZ is already heavily focused on individual behaviour change [47]. Therefore, any implementation of the proposed solutions should be carefully monitored to ensure the effects are as intended and equitable.

This GMB study is part of a larger project to operationalise systems thinking methods in combination with public health nutrition to achieve a healthy, sustainable food environment in NZ [21]. A related paper has detailed semi-structured interviews with national food-system stakeholders in NZ, collecting their views on the barriers and possible solutions to low FV intake in children [48]. In comparing the two studies, community workshop participants were clearer about the dominant role that fast food plays in children’s nutrition, displacing FV intake, whereas the national stakeholders tended to focus on the role of parents and/or schools and government to provide access to healthy food. Interestingly, the solutions proposed by workshop participants and the national stakeholders were similar, with a focus on changing individual behaviours through nutrition education and the school food environment. However, the national stakeholders were also able to suggest potential ‘upstream’ actions [48]. In both studies, it would have been useful to include an additional session with participants where researchers could present the international evidence on the efficacy and equitable nature of the actions proposed.

### Strengths and limitations of the study

This project was based on an authentic partnership between the university researchers and HFW, relying on the strengths and expertise of each team member, and undertaken with a non-judgemental and collaborative mind-set. Aspects which fostered the partnership were: the project manager being employed by the University but based at HFW, multiple face-to-face planning sessions where roles for the workshops were clearly defined, check-ins after each workshop, and regular email communication.

GMB proved a useful method to engage and actively involve a wide range of participants to address a public health nutrition issue that the community themselves would not necessarily have identified or prioritised. The workshops were lively and the enthusiasm and attention of participants was strongly evident. Holding multiple workshops each separated by one week allowed participants to think about the issue and come back with new ideas. Furthermore, the participatory aspect of GMB promoted greater ownership of the actions. The workshops succeeded in catalysing community action on children’s FV intake, and local actions are now being co-designed by the research participants, with oversight and management from HFW. The University researchers and HFW are expertly positioned to catalyse action at multiple levels which may be more difficult for the community to operationalise (e.g. reducing fast-food advertising to children and the density of fast-food outlets).

An important limitation of this study is the potentially narrow generalisability of the findings, both to communities around NZ and worldwide. The small number of participants were chosen purposively and via snowballing. Most participants were strongly involved in their community and had varying degrees of influence and the ability to enact many of the changes which were proposed at the school and community-level. Attempts to recruit community participants beyond the relationships held by HFW were largely unsuccessful, in part due to the time commitment required and uncertainty about what the workshops would entail. Consequently, the views of participants on the barriers and solutions to children’s low FV intake may not be representative of the wider community. Readers should also be aware that 70% of the workshop participants were female, including all of the parents, and it would have been preferable to gather fathers’ perspectives on this topic as well.

## Conclusions

The lens of system dynamics (SD) applied in this study, and the process of GMB, provided a new way of thinking about children’s low and inequitable FV intake. The method effectively enabled participants to identify several systemic barriers in their community which they believed required action, in particular, the proliferation of fast-food outlets and unhealthy food marketing. However, the shift from considering barriers to proposing actions in the system, whereby participants envisage ways to modify the underlying norms and goals of the system, takes considerable time and trained SD practitioners. Future researchers wishing to use GMB could use the template provided in this research to structure their GMB sessions, but would be advised to allocate an additional step to co-design potential actions in detail with participants and specify how the action will influence the system as represented in the CLD. This research has identified several potential actions now ready for further development and testing in the local community. GMB has proved a useful method to explore community views on a complex issue and catalyse action to improve public health nutrition.

## Supporting information

S1 Table COREQ (COnsolidated criteria for REporting Qualitative research) checklist. (PDF)

S1 Fig Graphs over time themes from Workshop 1. (PDF)

S2 Fig Linear map from Workshop 1. (PDF)

S3 Fig Spheres of influence from Workshop 3. (PDF)

S4 Fig Prioritising action matrix from Workshop 3. (PDF)

## Abbreviations

CLD: causal loop diagram
FV: fruit and vegetable
GMB: group model building
HFW: Healthy Families Waitākere
NZ: New Zealand
SD: System dynamics

## References

[1] Ministry of Health. Food and Nutrition Guidelines for Healthy Children and Young People (aged 2–18 years): a background paper. Wellington: Ministry of Health; 2012.

[2] Ministry of Health. Annual Data Explorer 2016/17: New Zealand Health Survey. Available from: https://minhealthnz.shinyapps.io/nz-health-survey-2016-17-annual-update. Accessed 11 Dec, 2018.

[3] Ministry of Health. Regional Data Explorer 2014–2017: New Zealand Health Survey. Available from: https://minhealthnz.shinyapps.io/nz-health-survey-2014-17-regional-update. Accessed 14 Dec, 2018.

[4] Appleton KM, Hemingway A, Saulais L, Dinnella C, Monteleone E, Depezay L et al Increasing vegetable intakes: rationale and systematic review of published interventions. Eur J Nutr 2016; 55: 869 10.1007/s00394-015-1130-8 26754302 PMC4819941

[5] Rasmussen M, Krølner R, Klepp K, Lytle L, Brug J, Bere E, et al Determinants of fruit and vegetable consumption among children and adolescents: a review of the literature. Part I: quantitative studies. Int J Behav Nutr Phys Act 2006;3(22). 10.1186/1479-5868-3-22 PMC1564033 16904006

[6] Te Velde S, Twisk J, Brug J. Tracking of fruit and vegetable consumption from adolescence into adulthood and its longitudinal association with overweight. Br J Nutr 2007, 98(2), 431–438. 10.1017/S0007114507721451 17433126

[7] Mikkilä V, Räsänen L, Raitakari O, Pietinen P, Viikari J. Consistent dietary patterns identified from childhood to adulthood: The Cardiovascular Risk in Young Finns Study. Br J Nutr 2005, 93(6), 923–931. 16022763 10.1079/bjn20051418

[8] Ledoux TA, Hingle MD, Baranowski T. Obesity prevention relationship of fruit and vegetable intake with adiposity: a systematic review. Obes Rev 2011; 12:e143–e150. 20633234 10.1111/j.1467-789X.2010.00786.x

[9] Institute for Health Metrics and Evaluation. Global Burden of Disease Study Compare Tool.VisHub 2018. Available from: https://vizhub.healthdata.org/gbd-compare/

[10] Matheson A, Walton M, Gray R, Lindberg K, Shanthakumar M, Wehipeihana N, et al Summative evaluation report of Healthy Families NZ: September 2018. Wellington: Massey University; 2018.

[11] Swinburn BA, Sacks G, Hall KD, McPherson K, Finegood DT, Moodie ML, et al The global obesity pandemic: shaped by global drivers and local environments. Lancet 2011; 378(9793):804–814. 10.1016/S0140-6736(11)60813-1 21872749

[12] Swinburn BA, Kraak VI, Allender S, Atkins VJ, Baker PI, Bogard JR, et al The Global Syndemic of Obesity, Undernutrition, and Climate Change: The Lancet Commission report. Lancet 2019; 393(10173):791–846 10.1016/S0140-6736(18)32822-8 30700377

[13] Hovmand PS. Community Based System Dynamics. DE: Springer Verlag; 2014.

[14] Brennan LK, Sabounchi NS, Kemner AL, Hovmand P. Systems thinking in 49 communities related to healthy eating, active living, and childhood obesity. J Public Health Manag Prac 2015; 21 Suppl 3:S69. 10.1097/PHH.0000000000000248 25828223

[15] Allender S, Owen B, Kuhlberg J, Lowe J, Nagorcka-Smith P, Whelan J, et al A community based systems diagram of obesity causes. PLoS One 2015;10(7):e0129683 10.1371/journal.pone.0129683 26153893 PMC4496094

[16] Gittelsohn J, Mui Y, Adam A, Lin S, Kharmats A, Igusa T, et al Incorporating systems science principles into the development of obesity prevention interventions: principles, benefits, and challenges. Curr Obes Rep 2015;4(2):174–181. 10.1007/s13679-015-0147-x 26069864 PMC4452216

[17] Owen B, Brown AD, Kuhlberg J, Millar L, Nichols M, Economos C, et al Understanding a successful obesity prevention initiative in children under 5 from a systems perspective. PLoS One 2018;13(3):e0195141 10.1371/journal.pone.0195141 29596488 PMC5875853

[18] Friel S, Pescud M, Malbon E, Lee A, Carter R, Greenfield J, et al Using systems science to understand the determinants of inequities in healthy eating. PLoS One 2017;12(11):e0188872 10.1371/journal.pone.0188872 29190662 PMC5708780

[19] Urwannachotima N, Hanvoravongchai P, Ansah JP. Sugar-sweetened beverage tax and potential impact on dental caries in Thai adults: an evaluation using the group model building approach. Sys Res Behav Sci 2018; 36(1).

[20] Waqa G, Moodie M, Snowdon W, Latu C, Coriakula J, Allender S, et al Exploring the dynamics of food-related policymaking processes and evidence use in Fiji using systems thinking. Health Res Policy Syst 2017;15(74):1–8. 28851398 10.1186/s12961-017-0240-6 PMC5575848

[21] Waterlander WE, Ni Mhurchu C, Eyles H, Vandevijvere S, Cleghorn C, Scarborough P, et al Food futures: developing effective food systems interventions to improve public health nutrition. Agric Syst 2018:124–131.

[22] Tong A, Sainsbury P, Craig J. Consolidated criteria for reporting qualitative research (COREQ): a 32-item checklist for interviews and focus groups. Int J Qual Health Care 2007;19(6):349–357. 10.1093/intqhc/mzm042 17872937

[23] Hovmand PS, Rouwette, Etiënne AJA, Andersen DF, Richardson GP, Kraus A. Scriptapedia 4.0.6.; 2013.

[24] Richardson GP, Andersen DF. Teamwork in group model building. Syst Dyn Rev 1995;11(2):113–137.

[25] Maani KE, Cavana RY. Systems thinking, system dynamics. Auckland, New Zealand: Pearson Education New Zealand; 2007.

[26] Friel S, Hattersley L, Ford L, O’Rourke K. Addressing inequities in healthy eating. Health Promot Int 2015;30:77–88. 26420812 10.1093/heapro/dav073

[27] Burkett, I. An introduction to co-design. Centre for Social Impact. Retreived from: https://www.yacwa.org.au/wp-content/uploads/2016/09/An-Introduction-to-Co-Design-by-Ingrid-Burkett.pdf Accessed 8 March, 2019

[28] Vandevijvere S, Mackay S, D’Souza E, Swinburn B. How healthy are New Zealand food environments? A comprehensive assessment 2014–2017. Wellington: Univeristy of Auckland; 2018.

[29] Sushil Z, Vandevijvere S, Exeter DJ, Swinburn B. Food swamps by area socioeconomic deprivation in New Zealand: a national study. Int J Public Health 2017;62:869–877. 10.1007/s00038-017-0983-4 28534060

[30] Boyland EJ, Nolan S, Kelly B, Tudur-Smith C, Jones A, Halford JC, et al Advertising as a cue to consume: a systematic review and meta-analysis of the effects of acute exposure to unhealthy food and nonalcoholic beverage advertising on intake in children and adults. Am J Clin Nutr 2016, 103(2): 519–533. 10.3945/ajcn.115.120022 26791177

[31] Sadeghirad B, Duhaney T, Motaghipisheh S, Campbell NRC Johnston BC. Influence of unhealthy food and beverage marketing on children’s dietary intake and preference: a systematic review and meta-analysis of randomized trials. Ob Rev 2016, 17: 945–959. 10.1111/obr.12445 27427474

[32] Roy H. About us: from the chair. Retrieved from: http://www.asa.co.nz/about-us/from-the-chair/. Accessed 16 January, 2019

[33] Advertising Standards Authority. Children and young people’s advertising code. Available from: http://www.asa.co.nz/codes/codes/new-children-young-peoples-advertising-code/. Accessed 16 Jan, 2019.

[34] Jaime PC, Lock K. Do school based food and nutrition policies improve diet and reduce obesity? Prev Med 2009; 48:45–53. 10.1016/j.ypmed.2008.10.018 19026676

[35] Ministry of Education. The National Administration Guidelines (NAGs). 2018; Available from: https://www.education.govt.nz/our-work/legislation/nags/. Accessed Jan 23, 2018.

[36] Micha R, Karageorgou D, Bakogianni I, Trichia E, Whitsel LP, Story M, et al Effectiveness of school food environment policies on children’s dietary behaviors: a systematic review and meta-analysis. PloS One 2018;13(3):e0194555 10.1371/journal.pone.0194555 29596440 PMC5875768

[37] Delgado-Noguera M, Tort S, Martínez-Zapata MJ, Bonfill X. Primary school interventions to promote fruit and vegetable consumption: a systematic review and meta-analysis. Prev Med 2011;53(1):3–9. 21601591 10.1016/j.ypmed.2011.04.016

[38] Watts C. External evaluation of Fruit in Schools final report. Wellington: Quigley and Watts 2018.

[39] Mackay S, Buch T, Vandevijvere S, Goodwin R, Korohina E, Funaki-Tahifote M, et al Cost and Affordability of Diets Modelled on Current Eating Patterns and on Dietary Guidelines, for New Zealand Total Population, Māori and Pacific Households. Int J Environ Res Public Health 2018, 15:1255 10.3390/ijerph15061255 PMC6025104 29899249

[40] Gerritsen S, Wall C. How We Eat–Reviews of the evidence on food and eating behaviours related to diet and body size. Wellington: Ministry of Health 2017.

[41] Dudley DA, Wayne G, Peralta LR. Teaching approaches and strategies that promote healthy eating in primary school children: a systematic review and meta-analysis. Int J Behav Nutr Phys Act 2015;12(28):1–26. 25889098 10.1186/s12966-015-0182-8 PMC4416340

[42] Hersch D, Perdue L, Ambroz T, Boucher JL. The impact of cooking classes on food-related preference, attitudes, and behaviors of school-aged children: A systematic review of the evidence, 2003–2014. Prev Chronic Dis 2014;11:1–10. 10.5888/pcd11.140267 PMC4222785 25376015

[43] Hodder RK, Stacey FG, O’Brien KM, Wyse RJ, Clinton-McHarg T, Tezelepis F, et al Interventions for increasing fruit and vegetable consumption in children aged five years and under. Cochrane Database for Systematic Reviews 2018 10.1002/14651858.CD008552.pub4 29365346 PMC6491117

[44] Flego A, Herbert J, Waters E, Gibbs L, Swinburn B, Reynolds J, et al Jamie’s Ministry of Food: Quasi-experimental evaluation of immediate and sustained impacts of a cooking skills program in Australia. PLoS One 2014;9(12):1–18. 10.1371/journal.pone.0114673 PMC4267737 25514531

[45] Frieden TR. A framework for public health action: the health impact pyramid. Am J Public Health Res 2010;100(4):590–595. 10.2105/AJPH.2009.185652 PMC2836340 20167880

[46] Adams J, Mytton O, White M, Monsivais P. Why are some population interventions for diet and obesity more equitable and effective than others? The role of individual agency. PLoS Medicine 2016 4 5;13(4):1–7. 10.1371/journal.pmed.1001990 PMC4821622 27046234

[47] Signal L, Ratima M editors. Promoting health in Aotearoa New Zealand. Dunedin, New Zealand: Otago University Press; 2015.

[48] Gerritsen S, Harré S, Swinburn B, Rees D, Renker-Darby A, Bartos AE, et al Systemic Barriers and Equitable Interventions to Improve Vegetable and Fruit Intake in Children: Interviews with National Food System Actors. Inter J Env Res Public Health 2019; 16:1387. 10.3390/ijerph16081387 PMC6518010 30999659

