# Supplementary material for: Improving low fruit and vegetable intake in children: Findings from a system dynamics, community group model building study
Source: PLoS One. 2019 Aug 15;14(8):e0221107. doi: 10.1371/journal.pone.0221107 (PMC6695127; doi:10.1371/journal.pone.0221107)
Supplement: S1 Fig — (PDF) [file pone.0221107.s002.pdf]

Eating F+V behaviour over time

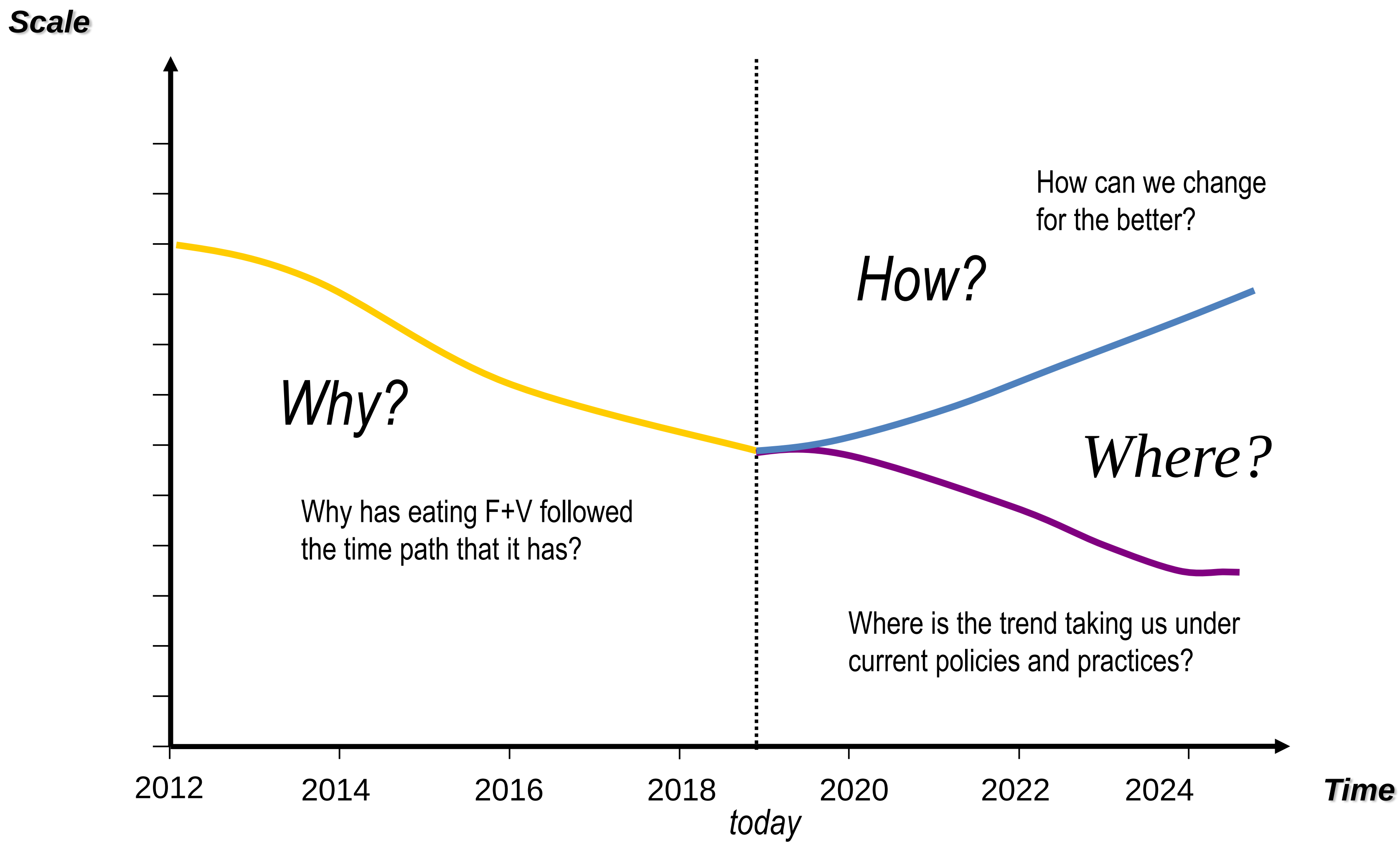

Key variables over time

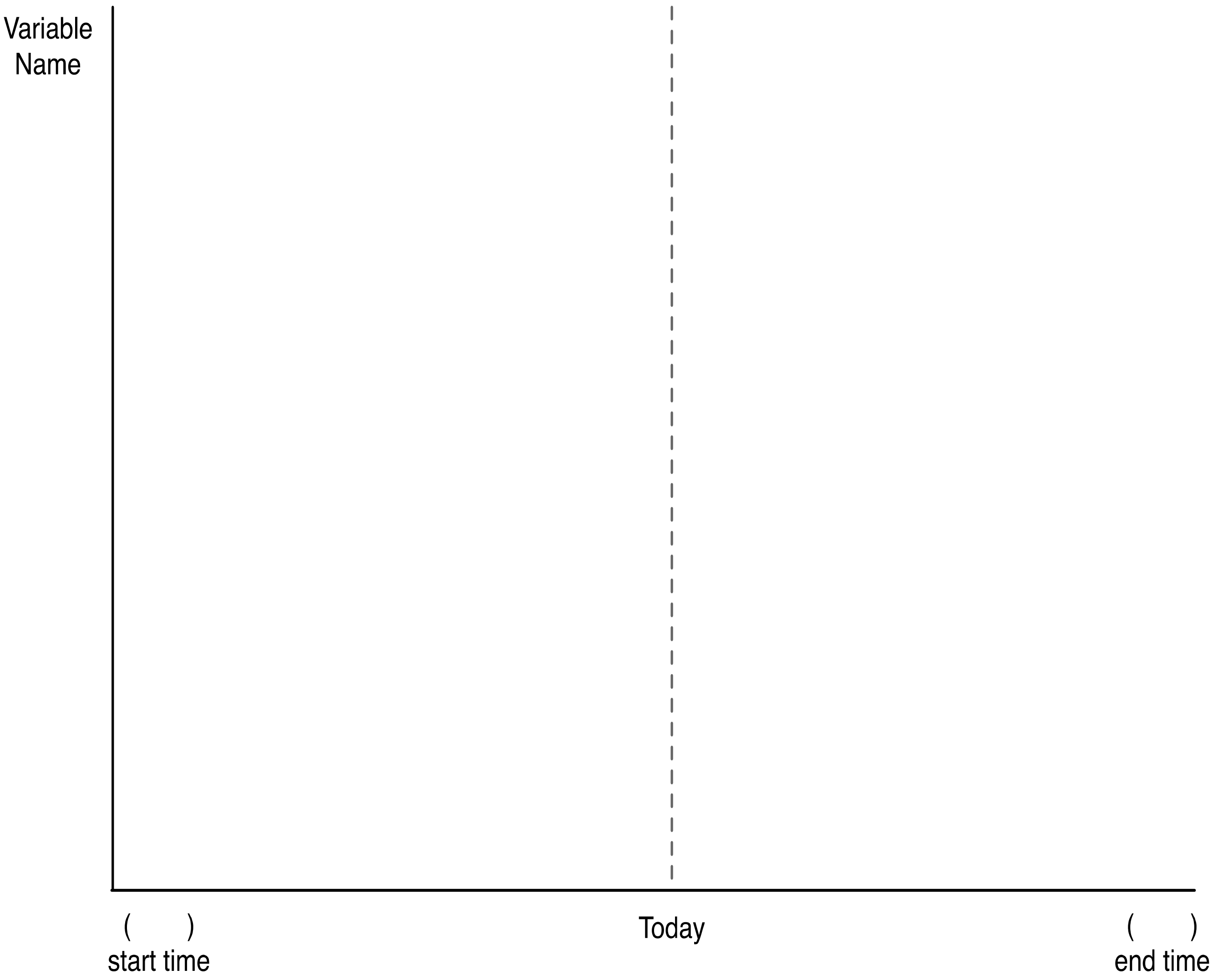

Access to fast-food / number of takeaway outlets in neighbourhood / fast-food options

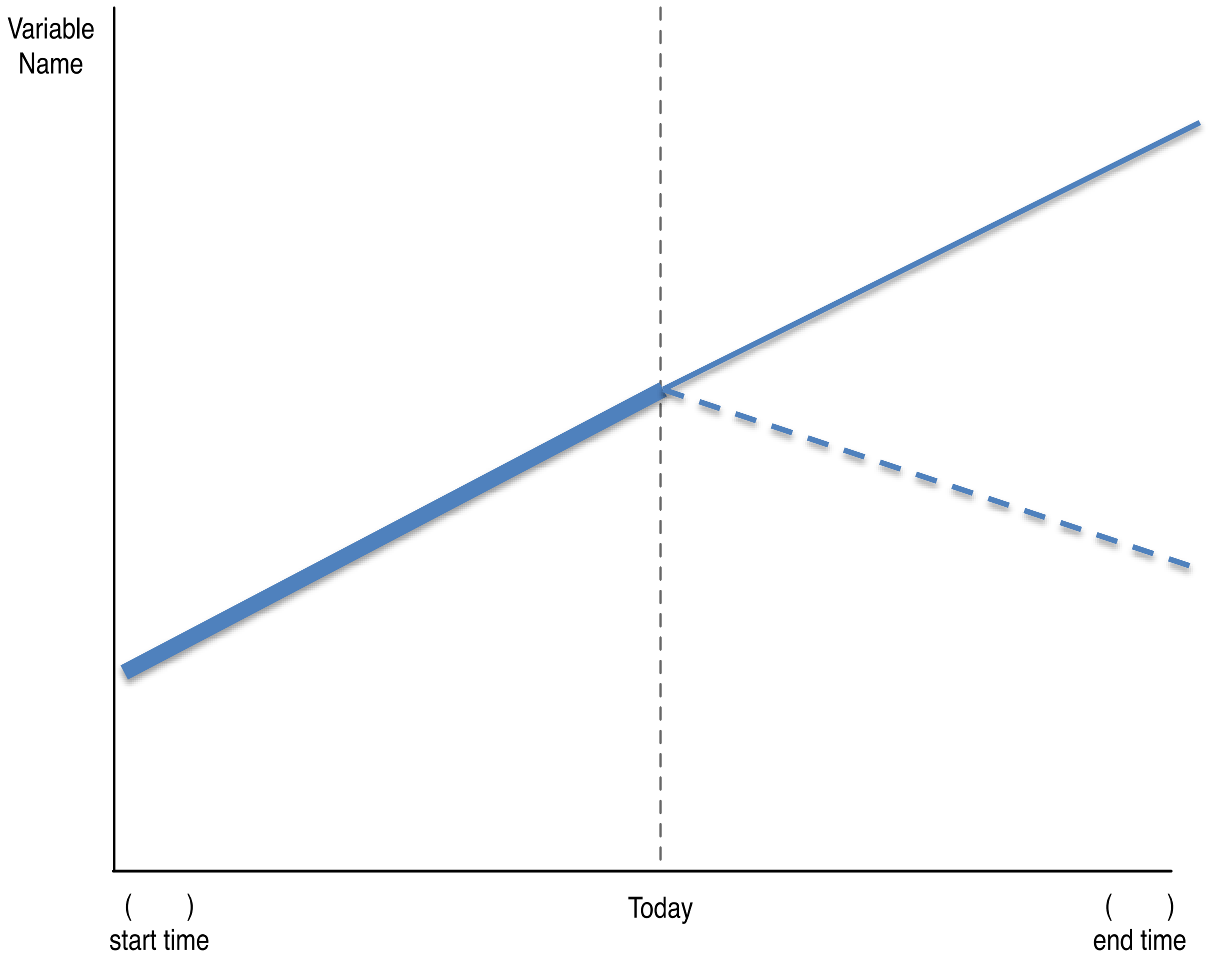

Marketing of fast-food (on TV, social media and also sponsorship)

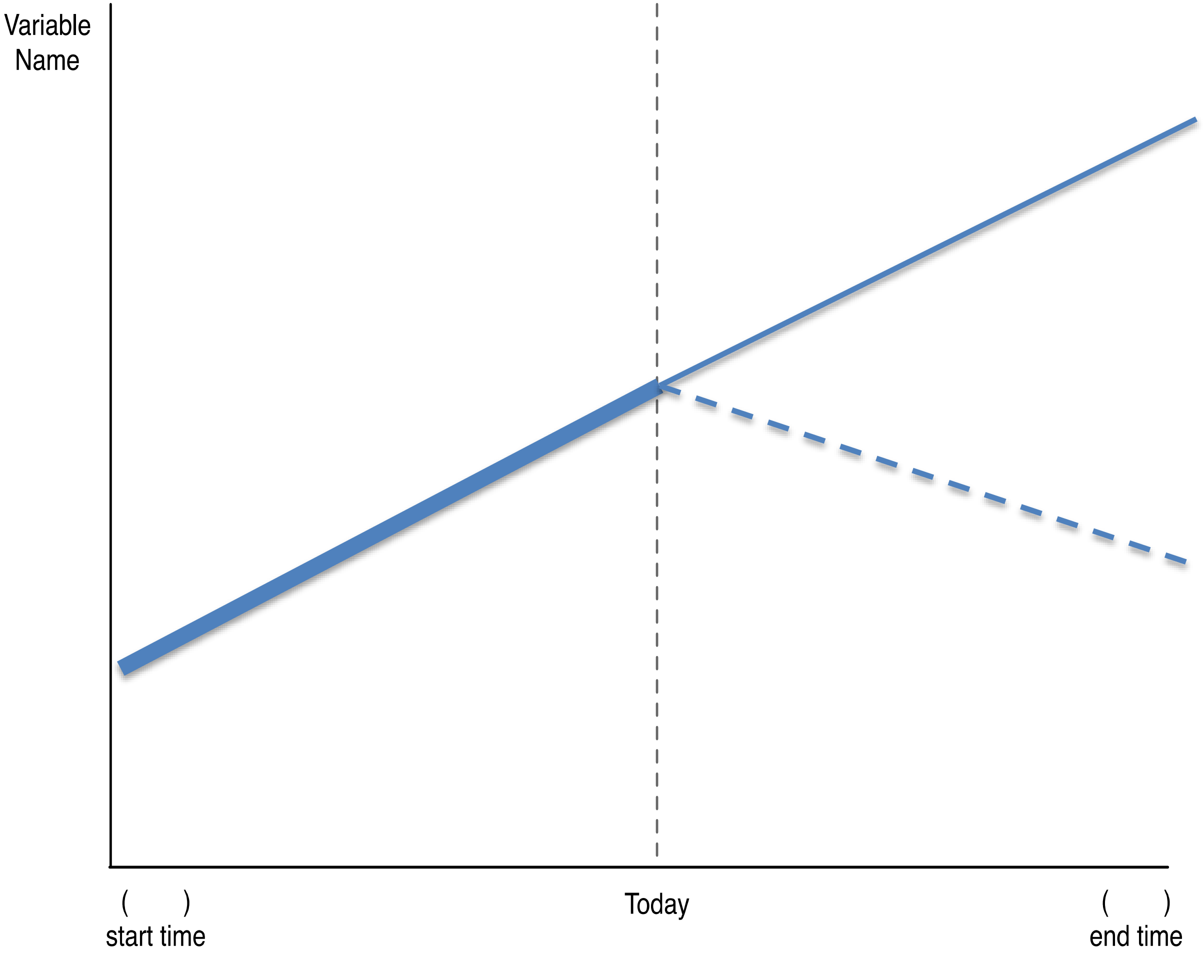

Price of vegetables (e.g. kumara, cauliflower, broccoli, onions, potatoes)

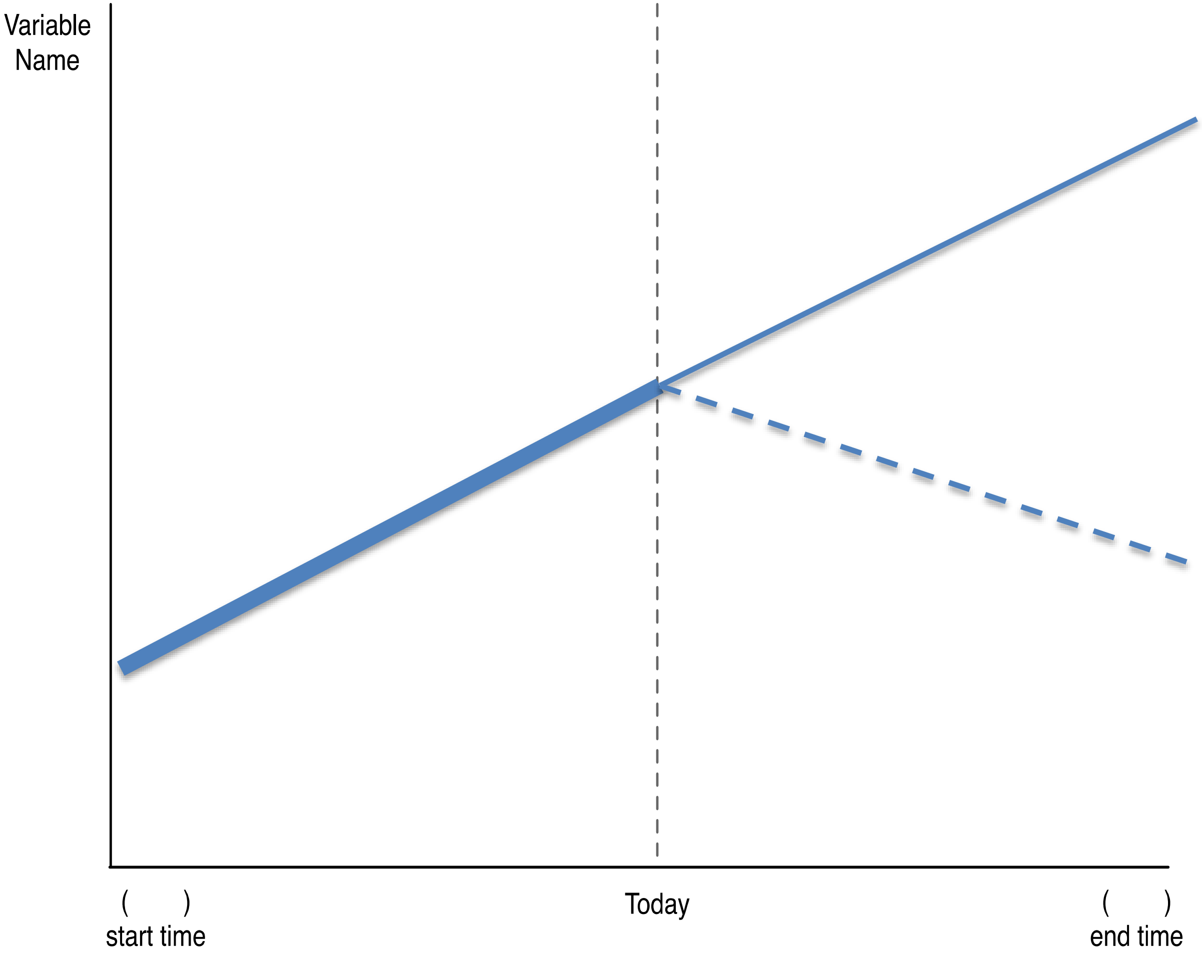

Price of fast-food (deals etc.)

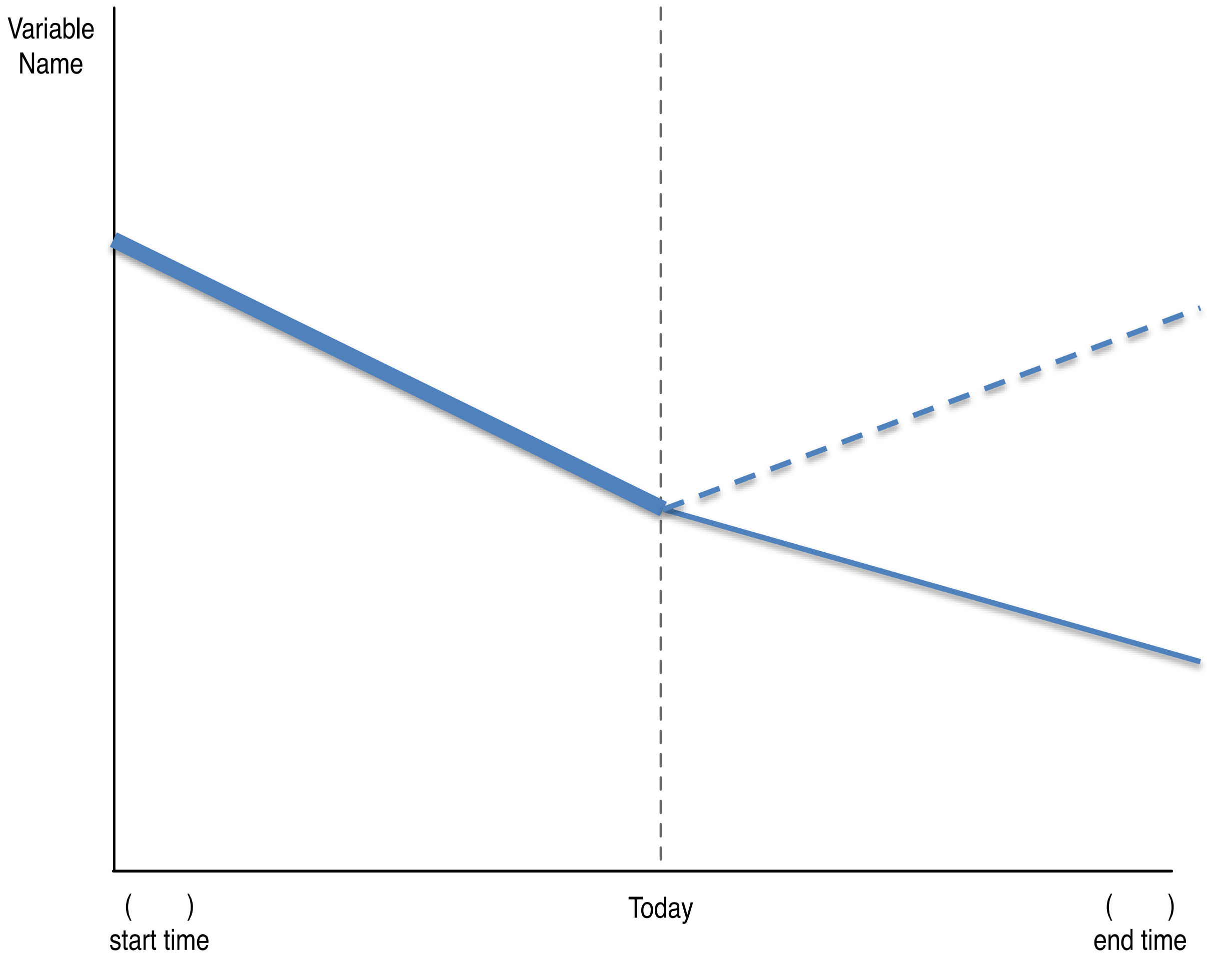

Work-life (low-income, two parents working, inflexible hours, high work load)

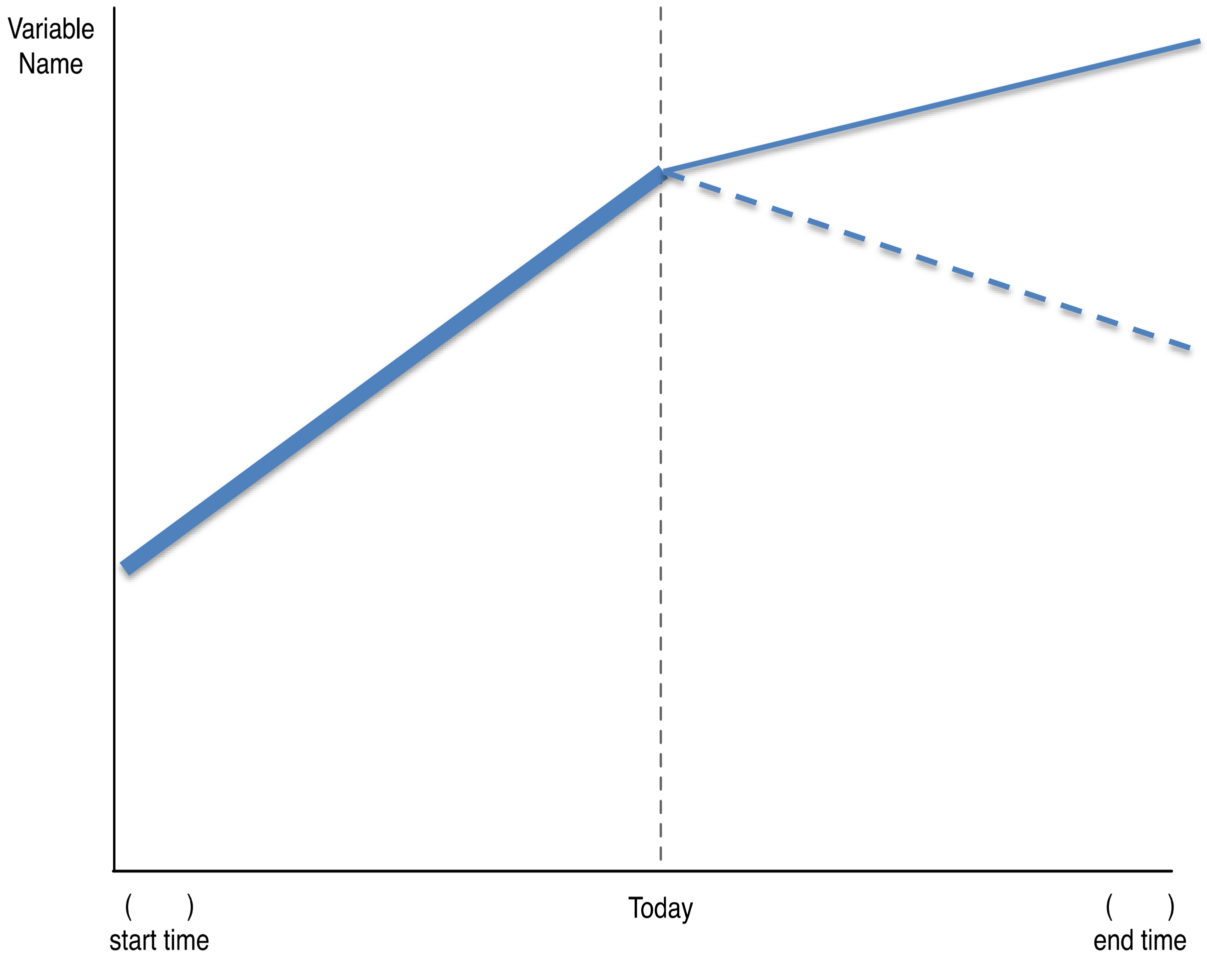

Nutrition knowledge (due to confusion about nutrition, misleading information spread)

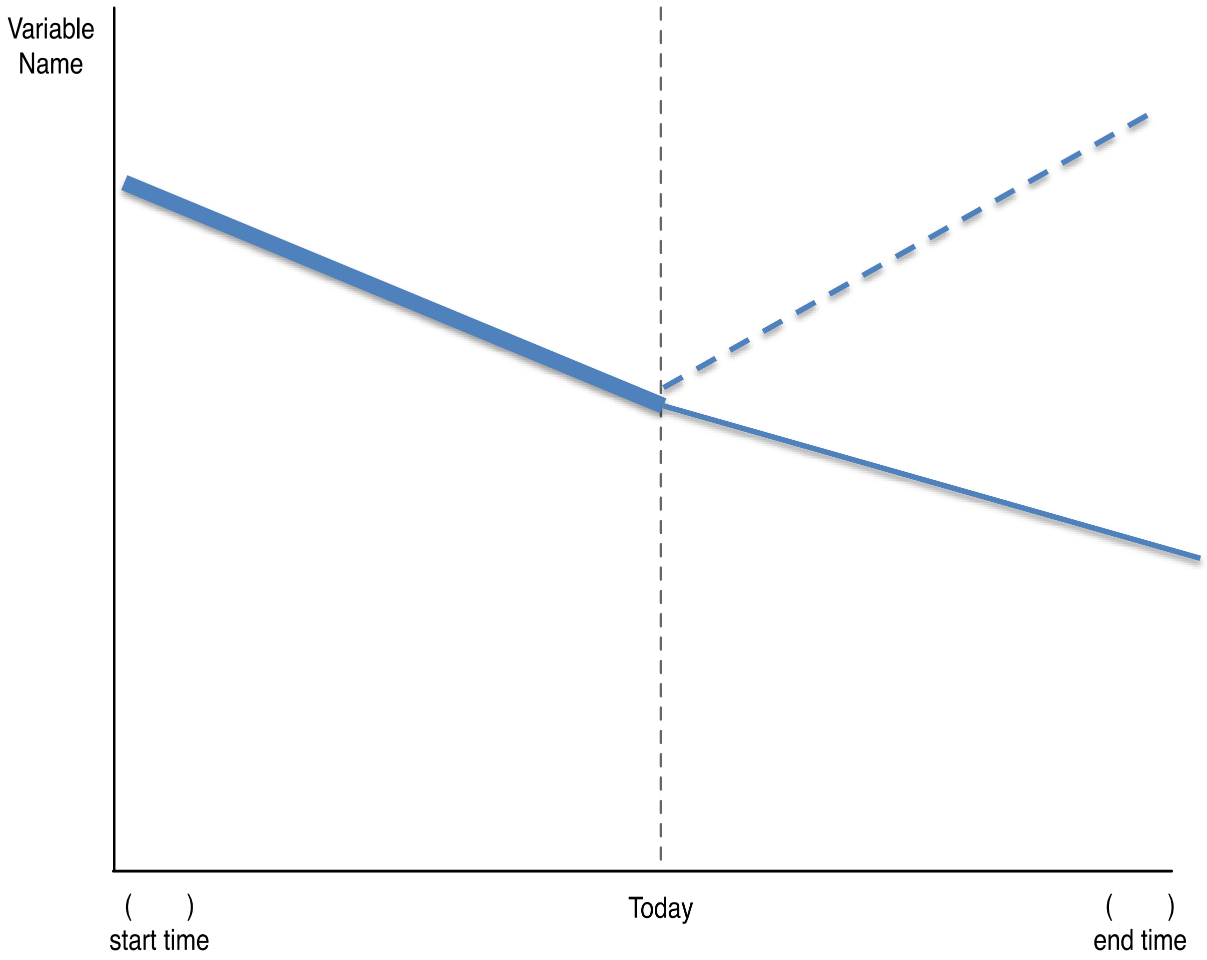

Growing own vegetables and fruit trees
(space to plant, access to gardens, sharing produce)

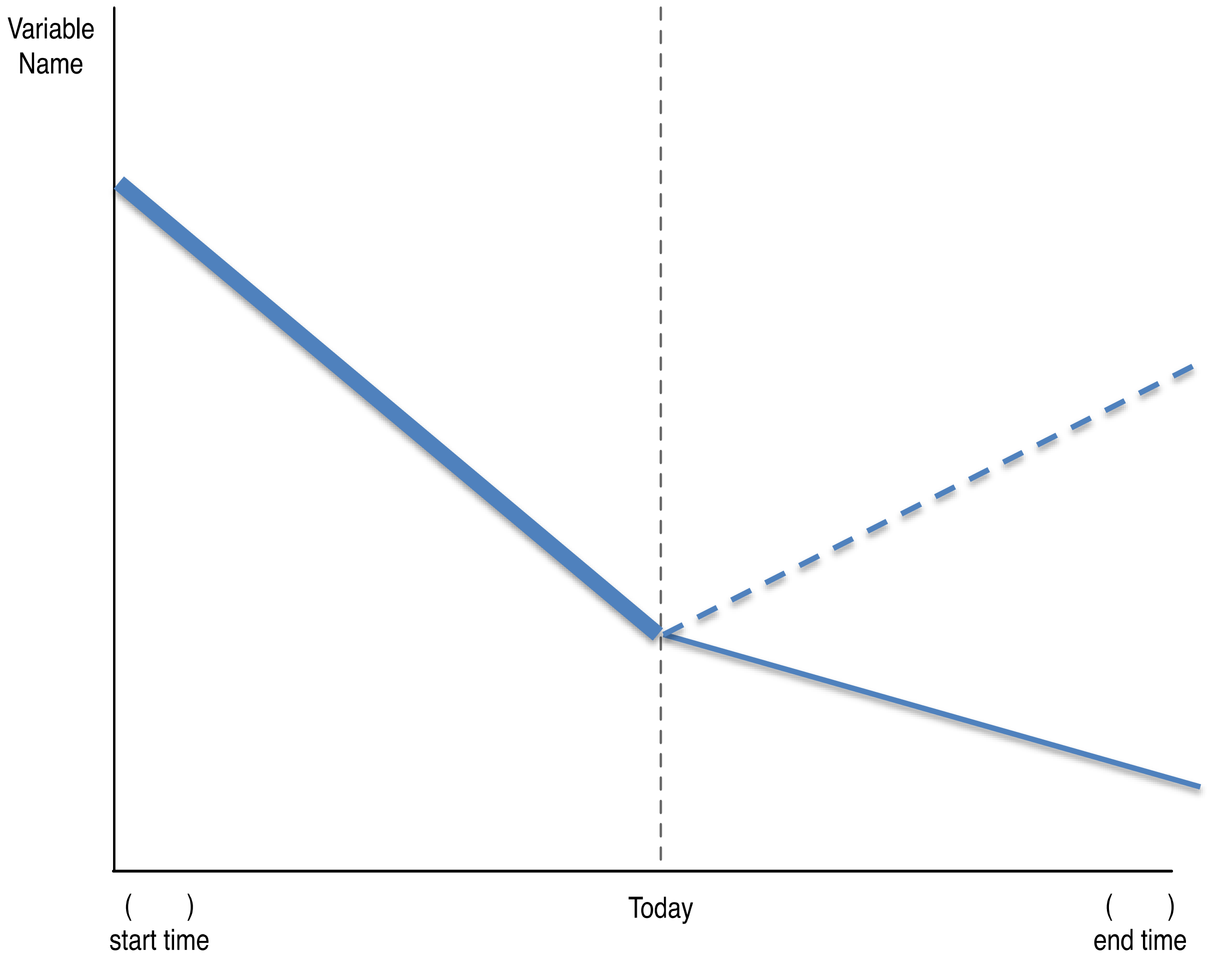

Other variables mentioned

- Peer pressure / social pressure (increase)
- Eating lunches from home everyday (decrease)
- More pocket money given to children
- Trust in growers e.g. pesticides (decreasing)
- Perceived quality of fruit and veg in supermarkets (decreasing)
- Supplement use

Some potentially positive trends

- Sampling / trying fruits and vegetables (increasing)
- Specialised food delivery with high fruit/veg (increasing)
- Cultural food options available in the community
