# Supplementary figures and images for: Improving low fruit and vegetable intake in children: Findings from a system dynamics, community group model building study
Source: PLoS One. 2019 Aug 15;14(8):e0221107. doi: 10.1371/journal.pone.0221107 (PMC6695127; doi:10.1371/journal.pone.0221107)

# CHILDREN'S F+V INTAKE

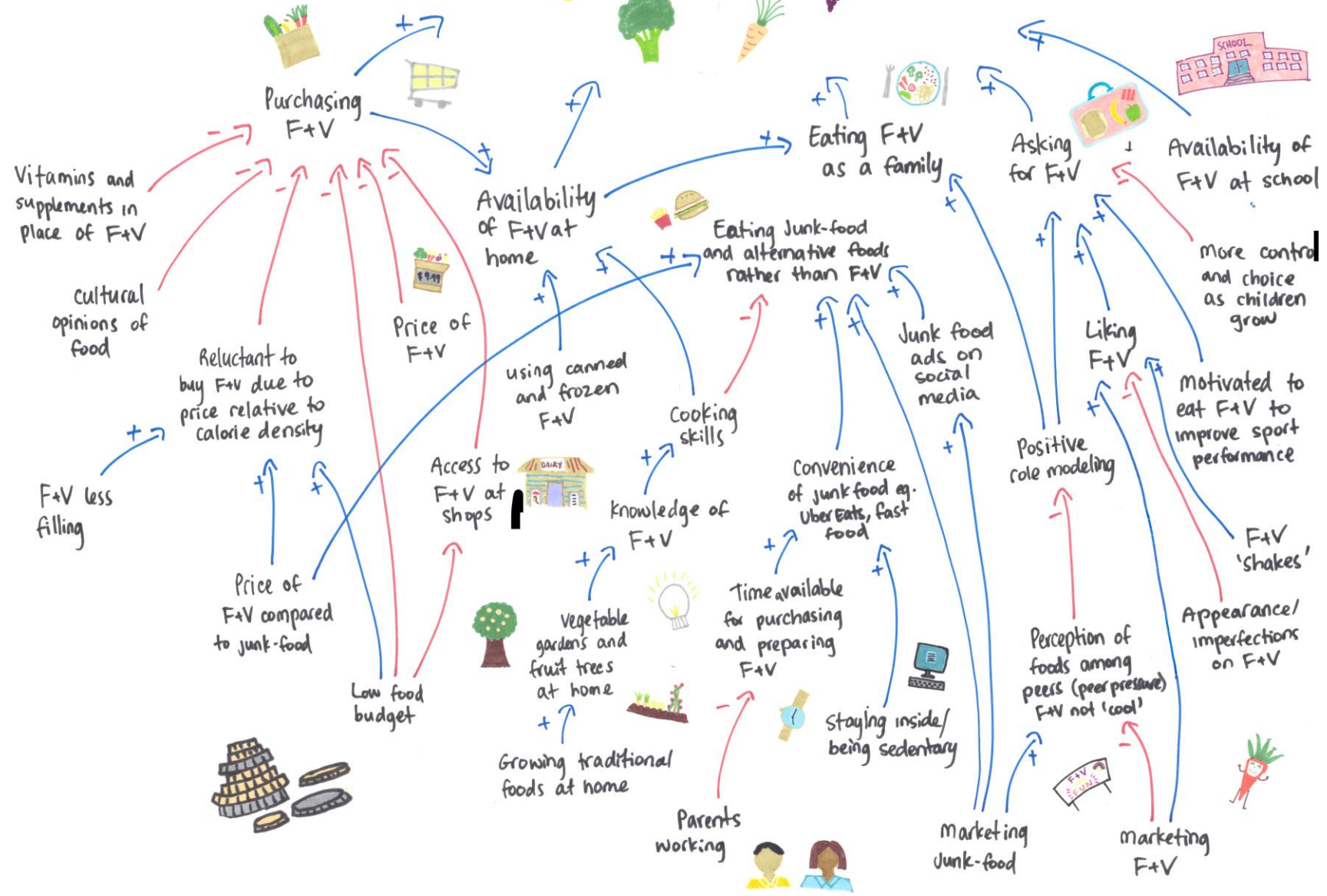

Supplement: S2 Fig — (PDF) [file pone.0221107.s003.pdf]

# Spheres of influence

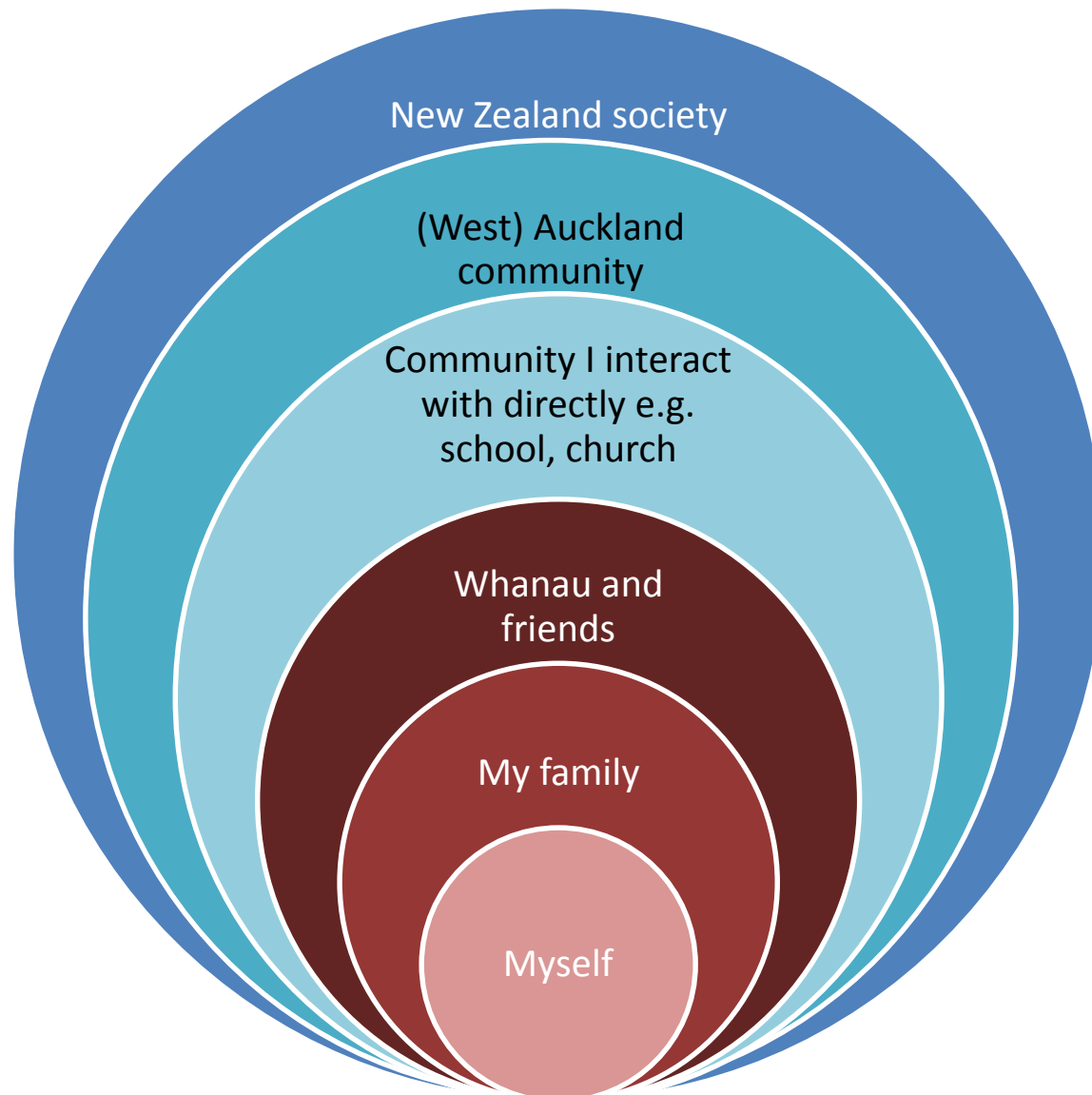

Supplement: S3 Fig — (PDF) [file pone.0221107.s004.pdf]

# Prioritising actions

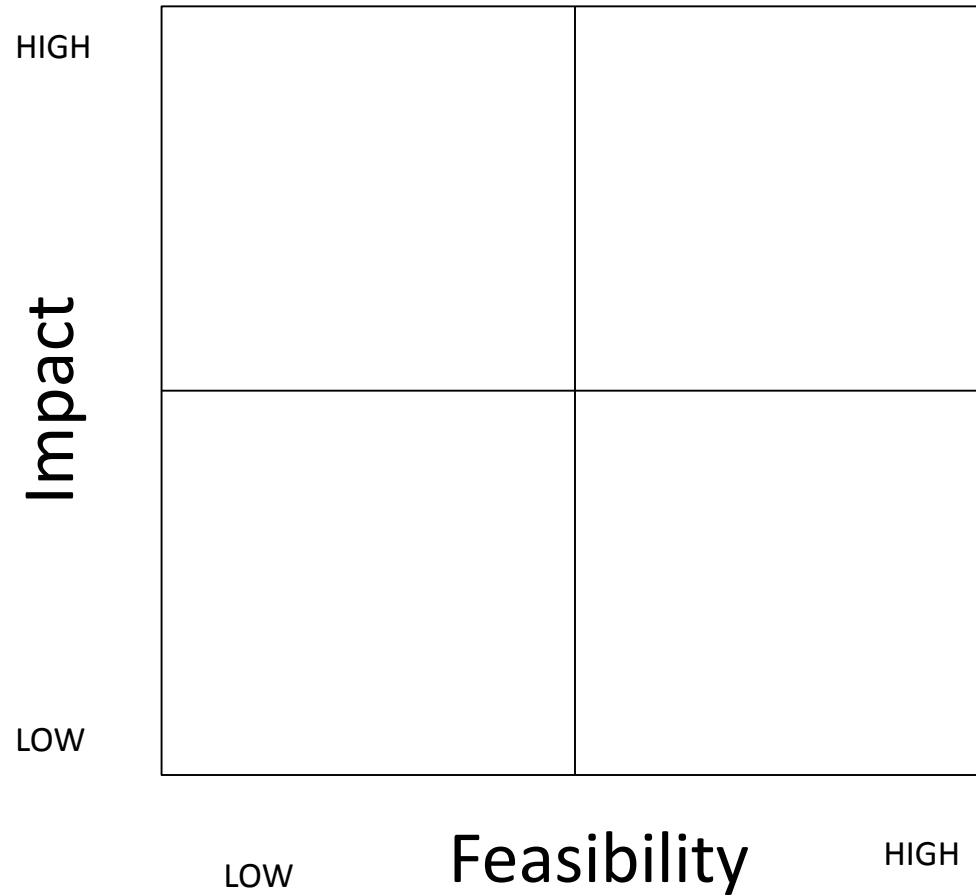

Supplement: S4 Fig — (PDF) [file pone.0221107.s005.pdf]
